# Infection status and risk factors of HIV, HBV, HCV, and syphilis among drug users in Guangdong, China - a cross-sectional study

**DOI:** 10.1186/1471-2458-10-657

**Published:** 2010-11-01

**Authors:** Jie Wu, Jinying Huang, Duorong Xu, Ciyong Lu, Xueqing Deng, Xiaolan Zhou

**Affiliations:** 1 Department of Medical Statistics and Epidemiology, School of Public Health, SUN Yat-sen University, Guangzhou, China; 2 Centers for Disease Control and Prevention, Qingyuan, China; 3 Department of Hematology, the First Affiliated Hospital, SUN Yat-sen, University, Guangzhou, China

## Abstract

**Background:**

China has witnessed a remarkable increase in sexually transmitted infections (STIs) and HIV. The study is to assess the prevalence of HIV, HBV, HCV and syphilis and related risk factors among drug users in mandatory detoxification center Qingyuan, Guangdong, China.

**Method:**

A cross-sectional study on drug use behaviors, sex behaviors, and presence of antibodies to HIV, HCV, Treponema pallidum, and surface antigen of HBV (HBsAg) was conducted among drug users recruited from 3 detoxification centers in Qingyuan, Guangdong, China. Risk factors for each of four infections were analyzed with logistic regression model.

**Results:**

A total of 740 subjects were recruited, the median age was 31 years old (range 24-38). The seroprevalence rates of HIV, HBsAg, HCV and syphilis were 4.6%, 19.3%, 71.6% and 12.6%, respectively. Risk factors for HIV were intravenous drug use and co-infection with syphilis. Having a regular sexual partner who was a drug user was considered to be a risk factor for HBV. Intravenous drug use was a risk factor for HCV. However, the consistent use of condoms with commercial sex partners was protective for HCV infection. Compared to drug users living in urban area, those living in rural areas were more likely to be infected with syphilis, and there was an association between commercial sex and syphilis.

**Conclusion:**

The prevalence of HIV, HBV, HCV and syphilis were high among drug users in detoxification centers in Qingyuan, thus, risk reduction programs for the drug user population is urgently required.

## Background

Since adopting a free market economy and an open door policy in 1978, China has witnessed a remarkable increase in sexually transmitted infections (STIs) and HIV [1]. By the end of 2007, the cumulative number of HIV positive individuals reported in China was 223,501, including 62,838 AIDS cases and 22,205 recorded deaths, 38.5% of reported HIV positive people were infected via intravenous drug use (IDU) [2]. Meanwhile, the prevalence of some blood-borne and sexually transmitted infections, including HBV, HCV and syphilis among drug users is also alarming. Several Studies in China have confirmed that the prevalence of hepatitis B virus (HBV), hepatitis C virus (HCV) and syphilis in drug user population is higher than that in the general population. For HCV, the prevalence varies from 14.3% for the general population to 91.6% among drug users, for HBV 8.3%compared to 79% and syphilis 1.4% compared to 29.2% [3-5].

The sharing of injecting equipment including needles and syringes is considered the major reasons for the extent of the HIV epidemic among the drug using population. Studies suggest that 54% of drug users inject and about 45% have recently shared needles and other injecting equipment [6,7]. Additionally, studies have reported high rates of commercial and unprotected sex among drug users [8,9]. Drug users are therefore regarded as a high risk group for HIV infection. There is also the potential for drug users who engage in risky behavior outside their home areas to transmit HIV to other drug users, their sexual partners, and others in the population [10]. Drug users are also at risk for some blood-borne and sexually transmitted infections including HBV, HCV, and syphilis.

HIV infection combined with certain STDs and blood borne infections can place a great burden on infected people. Numerous studies have shown that the clinical manifestations of certain STIs facilitate the transmission of HIV [11,12]. For example, in syphilis, the presence of a chancre provides a mechanical break in the protective skin barrier, allowing exposure to HIV. This is amplified by the increased number of inflammatory cells, the target of HIV, recruited to the syphilitic ulcer, as well as increased expression of HIV from those cells already infected [13,14]. Meanwhile, HIV infection, in turn, may increase the acquisition of other STIs and alter the natural history and response to standard therapy for ulcerative STIs, resulting in an ''epidemiological synergy'' between HIV and other STIs [15]. A growing body of evidence indicates that HIV-positive individuals are more likely to be infected with hepatitis B virus (HBV) than HIV-negative individuals, possibly as a result of shared risk factors. In addition, it is evident that immunosuppression brought about by HIV infection may cause reactivation or reinfection in those previously exposed to HBV [16]. It is also confirmed that HIV infection has a substantial impact on mortality among HCV-infected individuals, mainly due to HIV-induced immunodeficiency [17].

Data on prevalence and risk factors of HIV, HBV, HCV, and syphilis infections among drug users in mandatory detoxification centers(MDCs) in China are scarce, especially in Guangdong province; therefore, discussion about blood-borne and sexually transmitted infections within drug users often requires extrapolation from data obtained from other provinces even from other countries. Overall assessment of the extent and risk factors of these blood borne and sexually transmitted infections among drug users in MDCs will facilitate decision-making about how to screen for these infections, prevent further spread of these infections, and provide appropriate care to infected drug users. Therefore, the present research aimed to identify HIV, HBV, HCV, and syphilis infection status and related risk factors among drug users in MDCs.

## Methods

### Study setting

The study was conducted in 3 MDCs in Qingyuan, which is located in the middle north of Guangdong province. Since its first HIV case reported in 1999, the city witnessed an alarming increase in HIV infection. The status of HIV infection among drug users in Qingyuan is more severe than that of Guangdong on average. From 2000 to 2001, the average prevalence of HIV among drug users was 3.38%, which is higher than that of the whole Guangdong province which reported 1.74% and 2.67% respectively [18]. Given other blood-borne viruses and sexually transmitted infections, such as HBV, HCV, and syphilis, share similar part of transmission modes with HIV, we have a strong reason to believe there is a high prevalence of these infections among drug users in Qingyuan. It is therefore important to obtain overall data on the prevalence of HIV, HBV, HCV, and syphilis and risk factors among drug users in Qingyuan.

### Study design and study population

This study used a cross-sectional study design and was conducted from May to June 2008 and from October to December 2008 among a sample of male and female drug users in all of 3 MDCs in Qingyuan, Guangdong province. In preparation for this study, outreach workers conducted focus groups with drug users to assess the feasibility of conducting research among drug users and met with local official leaders to gain support for the research. Drug users in all 3 MDCs were assessed for eligibility and interests in participation by the physician in each MDC. To be eligible for the study, drug users had to (1) be able to provide written informed consent, (2) be at least 18 years of age, and (3) had a history of drug use. Subjects who had serious physical or mental illness or intelligence deficits were excluded. A total of 756 drug users were eligible for the study, 740 agreed to participate. Special precautions were taken to ensure that all eligible subjects understand that potential risks and benefits of study participation, and understand that participation was voluntary. Following a signed informed consent, Qingyuan CDC staff members surveyed all study participants in face-to-face interviews. Study protocol protected confidentiality through the use of personal identification numbers. After completion of the interview, subjects were asked to donate a blood specimen for research purposes. Prior to blood donation, Subjects were given pre-test counseling as well as information about how to obtain test results. Approximately one week after specimen collection, participants were given their HIV test results by a trained counselor during HIV post-test counseling. All interviewers, the physicians who screen for eligible participants and outreach workers received training in good clinical practice (GCP), human protection, and safeguarding confidentiality. No supervisor of MDCs including the physicians took part in the processes of obtaining informed consent and collecting data. The study received approval from the Institutional Review Board of Qingyuan CDC.

### Measurements

The interviews were developed after reviewing existing literature on risk factors for HIV, HBV, HCV and syphilis infection. We selected explanatory variables that had previously been identified as implicated in the transmission of HIV, HBV, HCV, and syphilis in this population: Injection of a mixture of drugs [3]; the age of initiating drug use [3]; ever injected drug (yes vs. no); [3] and syringe sharing (yes vs. no) [7]; frequency of injecting drug use [7]; duration of injecting drug use and/drug use [7]. We also included consistent condom use with non-regular sexual partners (yes vs. no) [4] and consistent condom use with commercial sexual partners (yes vs. no) [4].As in previous work, consistent condom use was defined as using condom every time in specific time frame. All variables can be found in table 1. In addition, we also added Infectious disease knowledge assessment to the questionnaire. Infectious disease knowledge assessment includes 7 questions concerning basic knowledge about HIV, 14 questions regarding the knowledge of HBV, HCV, and syphilis infections, 8 questions are about the preventive methods of 4 types infections. All of the above questions in the knowledge assessment were selected by a panel of consultants from the national behavioral and biological sentinel questionnaire in China [15] and other literature [3,4,7] about blood-borne and sexually transmitted disease.

**Table 1:** HIV, HBV, HCV, and syphilis infection status according to different variables

Variable	HIV(+/-)	t(Z)orχ^2^	HBsAg (+/-)	t(Z)orχ^2^	HCV (+/-)	t(Z)orχ^2^	syphilis(+/-)	t(Z)orχ^2^
Sex:								
male	31/648	0.01	129/550	0.56	493/186	3.93*	76/603	14.16*
female	3/58		14/47		37		17/44	
Age(year)								
Δ	34.9/31.1	3.15 *	30.6/31.4	-1.31	31.7/30.2	2.78*	32.4/32.1	1.69
Ethnicity:								
Han	34/696	0.00	142/588	0.56	525/205	2.33	90/640	2.80
Minority	0/10		1/9		5/5		3/7	
Marital status:								
single	9/325	6.87 *	51/283	6.44*	226/108	6.94*	42/292	5.84
Married or cohabitation	18/311		75/254		241/88		35/294	
Divorced or separated	7/70		17/60		63/14		16/61	
Education:								
<6 years	13/278	2.75	62/229	1.34	220/71	4.15	42/249	4.50
6 years-9 years	20/347		65/302		251/116		37/330	
Above 9 years	1/81		16/66		59/23		14/68	
Occupation:								
worker	6/155	1.23	39/122	12.19*	113/48	1.75	19/142	2.62
farmer	7/111		26/92		85/33		14/104	
unemployed	12/218		30/200		168/62		30/192	
driver	4/93		15/82		65/32		16/81	
others	5/129		33/101		99/35		14/128	
Monthly income:								
<1000 Yuan	14/435	7.35	86/363	0.48	322/127	12.57*	54/395	5.70
1000~2000 Yuan	12/172		34/150		137/47		21/163	
2000~3000 Yuan	3/55		13/45		31/27		13/45	
Above 3000 Yuan	5/44		10/39		40/9		5/44	
Registered Residence:								
Qingyuan	31/602	0.91	122/511	0.01	462/171	4.00*	76/557	1.25
Other regions	3/104		21/86		68/39		17/90	
Origin of family:								
unban	19/359	1.21	78/297	1.49	268/107	0.51	40/335	13.09*
Sub-urban	12/239		47/204		183/68		46/205	
city	3/111		18/96		79/35		7/107	
Knowledge score								
Δ	13.7/14.1	0.34	13.9/14.1	-0.29	14.1/14.0	0.29	14.0/14.1	-0.08
Total numbers of admission to MDC Δ	2.0/2.0	0.07	2.0/2.0	0.43	2.0/2.0	3.55*	2.0/2.0	2.01*
Age at first drug use Δ	26.7/23.5	2.71 *	23.1/23.1	-1.35	23.8/23.3	0.74	24.0/23.6	0.54
Duration of drug use:								
≥ 5 years vs. <5 years	7.89/7.73	0.76	6.1/8.0	0.50	7.9/5.4	2.79*	9.8/7.5	1.31
Ever used combined drugs:								
Y	8/232	1.28	47/193	0.01	169/71	0.25	37/203	2.62
N	26/474		96/404		361/139		56/444	
Continued table 1								
Ever injected drug in past 12 months before entering MDC:								
Y	33/519	9.49 *	105/447	0.12	427/125	35.14*	75/477	2.05
N	1/187		38/150		103/85		18/170	
Age at first injection Δ	27.1/25.0	1.81	25.4/25.2	0.32	25.3/24.9	0.66	24.7/25.3	-0.85
Duration of injection Δ	6.0/6.0	0.03	5.0/6.0	1.32	7.0/6.0	0.31	6.0/7.0	0.74
Frequency of injection in past 3 months before entering MDC:								
≤1time/day	13/269	2.19	55/227	0.20	218/64	5.38	35/247	2.44
2~3 time/day	17/206		42/181		177/46		31/192	
≥4 time/day	3/44		8/39		32/15		9/38	
Ever shared needle and/or syringe in the last injection:								
Y	7/128	0.20	30/105	1.18	106/29	0.13	22/113	1.11
N	26/391		75/342		321/96		53/364	
Ever shared need and/or syringe in last 3 months before entering MDC:								
Y	14/191	0.42	46/159	2.47	164/41	1.30	24/181	0.98
N	19/328		59/288		263/84		51/296	
regular sex partners ever used drug:								
Y	0/29	0.92	11/18	4.79*	19	1.39	8/21	6.90*
N	29/445		98/376		357		53/421	
Consistent condom use with regular sex partner in past 3 months before enteringMDC:								
Y	6/111	0.01	25/92	0.01	84	0.75	20/97	2.74
N	17/298		66/249		239		35/280	
Consistent condom use with non-regular sex partner in past 3 months before entering MDC:								
Y	4/86	0.00	23/67	5.64	57	1.78	11/79	0.08
N	5/113		15/103		85		16/102	
ever had commercial sex in past 12 months before entering MDC:								
Y	11/157	1.89	32/136	0.01	115	1.07	37/131	17.68
N	23/549		111/461		415		56/516	
Consistent condom use with commercial sex partner in past 3 months before entering MDC:								
Y	2/61	1.09	5/58	8.07 *	32	15.71*	12/51	0.33*
N	9/96		27/78		84		24/81	

Continued table 1

Note: * p < 0.05, (+/-): (the number of positive/the number of negative) Δ: mean or average

MDC: mandatory detoxification center;

Each participant provided blood specimens for anonymous testing for the presence of antibodies to HIV, HCV, Treponema pallidum, and HBV surface antigen (HBsAg). The methods of testing used HIV Antibody Test Kit and Sandwich ELISA (Beijing BGI-GBI Biotech Company, Beijing, China). All HIV positive blood samples were sent to HIV confirmatory laboratory in Guangdong Center for Disease Control and Prevention for confirmation. In accordance with the manufacturer's instructions, Antibody Test Kits were used for the presence of antibodies to HCV (Shanghai Rongshen Biotech Company, Shanghai, China), antibodies to Treponema pallidum (Beijing BGI-GBI Biotech Company, Beijing, China), and HBsAg (Beijing Wantai Biological Medicine Company, Beijing, China) respectively.

### Statistical analysis

The database was constructed with Epidata 3.0. SPSS version 11.0 was used for data analysis. Proportion, ratio, and median were applied to describe socio-demographic characteristics of participants and infection status of HIV, HBV, HCV, and syphilis. univariate analyses, such as Student's T-test, nonparametric tests, analysis of variance (ANOVA), and chi-square analysis were used to identify independent risk factors associated with positive test results of each infection. All risk variables detected by univariate analyses and variables reported by other literatures as risk factors were fitted into multivariate logistical regression analyses to determine risk factors. Odd ratios (ORs) and the corresponding 95% confidence intervals (95% CI) were used to compare proportions in independent groups of categorical sociodemographic, sexual, and drug use behavioral variables. A P value of <0.05 was considered as statistical significance.

## Result

### Socio-demographic characteristics

A total of 756 eligible drug users have been identified, and eventually 740 eligible drug users agree to participate in the study. 16 subjects refuse to participate because of weak health status (self-reported) or their lack of interests in this study. Of the 740 participants, there were 679 (91.8%) men; The median age was 31 years old (range: 24-38 years); 85.5% were local residents and 88.9% had received less than high school education; and 45.1% were single, 44.5% married, and 10.4% separated.

### Drug use and sexual behaviors

The age at the first drug use ranged from 17 to 30 years, the median age was 23 years.74.6% of subjects had injected drugs. of the participants who had injected drugs, 62.5% of injecting drug user had injected drugs for ≥ 5 years. 37.1% had shared needle and/or syringe with others in the past 3 months before entering MDC. 68.0% subjects reported having sex with regular sex partners in the last 12 months before entering MDCs, whereas only 9.5% of study subjects reported using condoms every time when they had sex with their regular partners. 30.68% reportedly had sex with non-regular partners in the past 12 months before entering MDCs. And only 16.4% of subjects who had non-regular partners reported consistent condom use in sex with their non-regular sex partners. 21.65% male participants had purchased sex and 34.4% female subjects reported being involved in providing commercial sex in the past 12 months before entering MDCs.

### Infectious diseases knowledge

The average score on the **Infectious Diseases **Knowledge Test was 14.1 ± 7.0 (range: 0-28). None of the responders achieved the maximum score (29).

### Prevalence of HIV, HBV, HCV, and syphilis

A total of 34 (4.6%) drug users tested positive for HIV, 143 (19.3%) subjects were identified as HBsAg positive, and the prevalence of HCV and syphilis were 71.6% and 12.6%, respectively. 82.2% of subjects have been exposed to at least one infection, 22.2% respondents reported two or more infections.

### Correlates of HIV, HBV, HCV, and syphilis infection

In univariate analysis, Drug users who were either divorced or separated, participants with older age, and those who reported having injected drugs in the past and older age of initiating drug use had significantly higher HIV infection rate(*p < 0.05)*. Syphilis infection also positively related to HIV infection. However, history of sharing needles, **Infectious Diseases **Knowledge score and consistent condom use with non-regular sex partner was not associated with HIV infection rate. The factors associated with HBV infection were being divorced or separated, occupation, drug use of regular sexual partner, consistent condom use in commercial sex (*p < 0.05)*. For HCV, the factors were gender, age, martial status, monthly income, registered residence, the total admissions to MDC, duration of drug use, ever having injected drugs in the past year before entering MDC, and consistent condom use in commercial sex(*p < 0.05)*. Participants who were female and those who are from rural areas, Drug users who report more admissions to MDC have reported significantly higher syphilis infection rate. The drug use of regular sexual partner and history of commercial sex in last year before entering MDC also positively associated with syphilis infection (*p < 0.05)*. (See table 1)

Table 2 shows the odd ratios (ORs) and corresponding 95% CIs of risk factors for HIV infection using multivariate regression analysis among 740 respondents. Among all variables which are significant (*p < 0.05) *in univariate analysis, intravenous drug use in the past year before entering MDC and syphilis infection were significantly associated with HIV infection. Table 3 shows the ORs and corresponding 95% CIs of risk factors for HBV infection. Only drug use of regular sexual partner was confirmed as a significant risk factor. Table 4 shows the results of multivariate regression analysis performed on HCV infection. Lower monthly income and intravenous drug use in the past year before entering MDC were risk factors, while consistent condom use in commercial sex was found to be a protective factor for HCV infection. Table 5 shows that the source of family, total admissions to MDC, and engaging in commercial sex in the past year before entering MDC are predictive factors for syphilis infection.

**Table 2:** Results of multivariate logistical regression for risk factor of HIV infection

Study variable	Wald χ^2^	P	OR	OR95%CI
Age	0.38	0.54	1.025	0.95~1.11
Marital status	0.98	0.61		
Unmarried	-	-	1.000	
Cohabitation/married/remarried	0.78	0.38	1.494	0.61~3.65
Separated/divorced/widowed	0.78	0.38	1.656	0.54~5.08
Mean age of initiating drug use	2.45	0.12	1.057	0.99~1.13
Ever injected drug in past 12 months before entering MDC: Yes vs.NO	6.54	0.01*	14.584	1.87~113.72
Syphilis co-infection: Yes vs. No	5.55	0.03*	2.713	1.18~6.22

Note: Variable in Brackets is Control. * p < 0.05 MDC: mandatory detoxification center;

**Table 3:** Results of multivariate logistical regression for risk factor of HBV infection

Study variable	Wald χ^2^	P	OR	OR95%CI
Marital status(unmarried)	1.47	0.48		
Cohabitation/married/remarried	0.09	0.77	0.82	0.21~3.19
Separated/divorced/widowed	1.39	0.24	0.22	0.02~2.74
Occupation (worker)	6.84	0.15		
farmer	1.33	0.25	0.31	0.04~2.26
Unemployed	4.27	0.04	0.14	0.02~0.90
Driver	0.48	0.49	0.58	0.13~2.70
others	4.48	0.03	0.13	0.02~0.86
Regular sex partners ever used drug: Yes vs. No	4.64	0.03*	5.13	1.16~22.73
Consistent condom use when having commercial sex in past 3 months before entering MDC: Yes vs. No	0.34	0.56	0.68	0.18~2.52

Note: Variable in Brackets is Control. * p < 0.05 MDC: mandatory detoxification center;

**Table 4:** Results of multivariate logistical regression for risk factor of HCV infection

Study variable	Wald χ^2^	P	OR	OR95%CI
Gender: female vs. male	2.36	0.12	3.93	0.69~22.49
Age	2.37	0.12	1.07	0.98~1.18
Marital status(unmarried)	5.41	0.07		
Cohabitation/married/remarried	0.69	0.41	0.60	0.18~2.02
Separated/divorced/widowed	2.75	0.10	3.81	0.78~18.58
Monthly income (< 1000)	15.01	< 0.01*		
1000~2000	5.28	0.02	4.96	1.27~19.44
2000~3000	7.19	< 0.01	0.16	0.04~0.61
3000 and more	0.35	0.55	1.73	0.28~10.56
Household register: Local vs. Non-Local	0.72	0.40	0.60	0.18~1.96
Total admissions to MDCs	1.33	0.25	1.16	0.90~1.49
Duration of drug use: ≥ 5 years vs. <5 years	3.13	0.08	0.91	0.81~1.01
Ever injected drug in past 12 months before entering MDC: Yes vs. No	5.02	0.03*	4.26	1.20~15.15
consistent condom use when having commercial sex in past 3 months before entering MDC: Yes vs. No	7.90	< 0.01*	0.28	0.12~0.68

Note: Variable in Brackets is Control * p < 0.05 MDC: mandatory detoxification center;

**Table 5:** Results of multivariate logistical regression for risk factors of syphilis infection

Study variable	Wald χ^2^	P	OR	OR95%CI
Gender: female vs. male	2.986	0.084	2.141	0.903~5.078
Family origin (urban)	7.660	0.022*		
Rural	6.083	0.014	3.996	1.329~12.012
Sub-urban	7.617	0.006	4.805	1.576~14.649
Total admissions to MDCs	10.587	0.001*	1.218	1.082~1.372
Regular sex partners ever used drug: Yes vs. No	1.740	0.187	1.926	0.727~5.100
Ever had Commercial sex in past 12 months before entering MDC: Yes vs. No	14.309	0.000*	3.210	1.754~5.874

Note: Variable in Brackets is Control * p < 0.05 MDC: mandatory detoxification center;

## Discussion

Drug use remains a concern in both developed and developing countries, and is a key factor for a myriad of other social problems. Drug using populations continue to demonstrate a significantly higher prevalence of blood-borne and sexually transmitted infections compared to general population [16,17], and there remains a need for more research regarding these diseases with this population. This study aims to report on the level of blood-borne and sexually transmitted infections and related risk behaviors in a sample of IDUs recruited from MDC in China.

Our study demonstrated a prevalence of HIV (4.6%), HBV (19.3%), HCV (71.6%), and syphilis (12.6%) in MDCs based drug user population. The HIV prevalence in our study was significantly higher than estimates from community-based surveys in other regions in China [15]. HCV infection is highly prevalent among Drug users in this city. It has been demonstrated that among Drug users, HIV infection is often introduced several years after HCV infection [18]. Populations with a high prevalence of HCV infection and low prevalence of HIV, such as that in this study, may reflect either harm-reduction programs which are effectively preventing the emergence of HIV, or a window of opportunity for the prevention of HIV infections among HCV-infected individuals. As is well known, China is a high prevalence area for HBV with a general seroprevalence of 9.8% [19]. A higher prevalence of HBV infection (19.3%) was identified among Drug users in this study. Our study found a lower syphilis seroprevalence of 12.6% compared to similar studies in Hubei province (59.5%) [5] and Lanzhou (17.5%) [20]. Further intervention targeting high risk populations is needed to contain the spread of these infections

Injecting drug use, needle/syringe sharing and unprotected sex are believed to be the major risk behaviors for HIV infection among the drug user population [21]. History of injecting drug and syphilis infection were identified as risk factors for HIV infection, which is consistent with a study conducted in Sichuan [22]. Syphilis infection is considered to be a signifier of high risk sexual behavior, and it is also an ulcerative disease which could facilitate transmission of HIV [11]. Condoms remain the most effective method of preventing HIV and syphilis transmission during sex and this study reinforces the need for effective promotion of condom use among the drug user population. However, our study failed to establish association between HIV infection and HIV knowledge level. This could be partly explained by the presence of re-education programs which cover HIV/AIDS knowledge in the MDC. So the knowledge level among all Drug users was improved after they entered the MDC.

various studies investigating female IDU in China and other countries have reported that about 21% to 60% were also engaged in sex work [23,24], and that female sex workers(FSWs) who were also IDUs (FSWIs) often engaged in behaviors involving risk of sexual transmission of HIV. In our study, female drug users also showed a high rate of involvement in commercial sex (34.4%). Female drug users are both at risk of infection with HIV and other sexually transmitted infections, and FSWIs also have the potential to transmit these infections to clients and others in the population and can therefore be regarded as a special high risk population. A primary focus of future risk reduction efforts should be placed on FSWs who were also IDUs (FSWIs).

Studies in other countries show the risk factors for syphilis among drug users are commercial sex, homosexual sex and multiple sexual partners [25,26]. In this study, commercial sex was confirmed as a risk factor for syphilis infections. It is interesting that drug users from rural areas demonstrated themselves being more susceptible to syphilis than those from urban areas, and this suggests us that future research designed to find more differences in syphilis prevalence between urban and rural areas, and reveal the possible reasons.

## Conclusion

High rates of intravenous drug use combined with high-risk sexual behaviors have resulted in the concentration of HIV, HBV, HCV, and syphilis infections in drug users. This highlights the importance of surveillance as a foundation for further interventions and research in the drug using population. The interventions that are most efficacious should be specifically designed for population segments, especially for sub-urban and rural areas. HIV and hepatitis co-infection among the drug users is an extremely serious issue and need particular attention. It is imperative to improve the coverage rate of overall intervention work such as extending the beneficiaries of the methadone maintenance treatment (MMT) program, which can reduce the rate of injecting drug use among community drug users and 100% condom use program in south China. The high prevalence of these blood borne and sexually transmitted infections in drug using populations in MDC suggests the urgent need for the introduction of some effective prevention strategies.

## Competing interests

The authors declare that they have no competing interests.

## Authors' contributions

CYL conceived the study, and helped to draft the manuscript. JW participated partly in design of study and performed data analysis besides mainly for manuscript draft. JYH and DRX are mainly for data collection, field control and data analysis. The rest of authors provided sufficient help for the study in terms of article revising and academic counseling. All authors read and approved the final manuscript.

## Acknowledgements

This document is an output from the cooperation of school of public health of SUN Yat-sen University and Qing Yuan CDC. During the period of study, thanks to the Qingyuan CDC provided substantial support to the project, we can complete the research successfully. Besides, we should especially thank Dr Jeffrey Grierson in Australian Research Centre in Sex, Health & Society; Faculty of Health Sciences Who helped to revise this manuscript in terms of language.

## Pre-publication history

The pre-publication history for this paper can be accessed here:

http://www.biomedcentral.com/1471-2458/10/657/prepub
